# Effectiveness of switching endoscopists for repeat surveillance colonoscopy: a retrospective study

**DOI:** 10.1186/s12876-023-02981-3

**Published:** 2023-10-06

**Authors:** Naoya Okada, Jun Arimoto, Takanori Nishiguchi, Mikio Kobayashi, Toshihiro Niikura, Hiroki Kuwabara, Michiko Nakaoka, Atsushi Nakajima, Hideyuki Chiba

**Affiliations:** 1 Department of Gastroenterology, Omori Red Cross Hospital, 4‑30‑1, Chuo, Ota-Ku, Tokyo 143‑8527 Japan; 2 Department of Gastroenterology and Hepatology, Yokohama City University School of Medicine, 3-9 Fukuura, Kanazawa-ku, Yokohama, Japan

**Keywords:** Colorectal neoplasia, Polyp detection, Surveillance colonoscopy, Adenoma detection rate

## Abstract

**Background:**

Surveillance colonoscopy decreases colorectal cancer mortality; however, lesions are occasionally missed. Although an appropriate surveillance interval is indicated, variations may occur in the methods used, such as scope manipulation or observation. Therefore, individual endoscopists may miss certain areas. This study aimed to verify the effectiveness of performing repeat colonoscopies with a different endoscopist from the initial procedure.

**Methods:**

We retrospectively reviewed a database of 8093 consecutive colonoscopies performed in the Omori Red Cross Hospital from January 1st 2018 to June 30th 2021. Data from repeat total colonoscopies performed within three months were collected to assess missed lesions. The patients were divided into two groups according to whether the two examinations were performed by different endoscopists (group D) or the same endoscopist (group S). The primary outcome in both groups was the missed lesion detection rate (MLDR).

**Results:**

Overall, 205 eligible patients were analyzed. In total, 102 and 103 patients were enrolled in groups D and S, respectively. The MLDR was significantly higher in group D (61.8% vs. 31.1%, P < 0.0001). Multivariate logistic regression analysis for the detection of missed lesions identified performance by the different endoscopists (odds ratio, 3.38; 95% CI, 1.81–6.30), and sufficient withdrawal time (> 6 min) (odds ratio, 3.10; 95% CI, 1.12–8.61) as significant variables.

**Conclusions:**

Overall, our study showed a significant improvement in the detection of missed lesions when performed by different endoscopists. When performing repeat colonoscopy, it is desirable that a different endoscopist perform the second colonoscopy.

**Trial registration:**

This study was approved by the Institutional Review Board of the Omori Red Cross Hospital on November 28, 2022 (approval number:22–43).

## Background

Colorectal cancer (CRC) is the third most common malignancy and the second most common cause of mortality worldwide [1]. Colonoscopy is considered the gold standard for CRC screening, and greatly decrease its incidence and mortality [2]. Appropriate surveillance colonoscopy has been shown to decrease colorectal cancer mortality [3]; however, lesions may occasionally be missed on colonoscopy as the colon has blind spots, caused by flexures and folds. Although an appropriate interval of surveillance is indicated [3], the exact methods applied, such as scope manipulation or observation, may vary among endoscopists. To further reduce the mortality rate of CRC, it is necessary to improve the quality of colonoscopy and to reduce the number of missed cancerous and precancerous lesions.

Adenoma detection rate (ADR) is considered the primary quality indicator in colonoscopy due to lower CRC incidence and mortality [4]. However, despite efforts to maintain a high ADR, CRC may occasionally develop during the interval between scheduled colonoscopies, which is called post-colonoscopy colorectal cancer (PCCRCs). The presence of PCCRCs may indicate the limitation of the ADR in terms of not assessing missed lesions [5]. Therefore, the adenoma miss rate (AMR) has drawn significant attention as a specific indicator of missed lesions. The AMR is estimated to be approximately 22% [6, 7], which can lead to PCCRC [8].

Reducing AMR is vital for decreasing the incidence of CRCs. Several factors, including instrumental, endoscopic, and patient factors, may cause missing lesions. With regard to instrumental factors, over the last decade, new techniques and many technological devices have been proposed to improve adenoma detection, such as blue light-based electronic chromoendoscopy [9]. In recent years, artificial intelligence has emerged as a standardized second observer, and Wang et al. reported reduced AMR with colonoscopy using a computer-aided detection system compared with conventional colonoscopy [10]. Relevant endoscopist factors included scope manipulation and observations. As colonoscopy is performed by humans, individual differences may exist to a certain extent. A study using eye-tracking technology revealed that the visual gaze patterns when watching a colonoscopy video varied from one endoscopist to another [11]. Several patterns of eye movement and scope manipulation have been observed during actual colonoscopies [12]. Furthermore, a previous report revealed that the polyp detection rate varies depending on visual gaze patterns and scope manipulation [13]. From these reports, individual differences in visual gaze patterns and scope manipulation may exist, and may be associated with polyp detection.

When performing repeated colonoscopies on the same patient, the polyp detection rate may change if a different endoscopist with a different gaze pattern and scope manipulation pattern performs the second colonoscopy. Therefore, we hypothesized that changing endoscopists may contribute to detecting lesions missed in the repeat colonoscopy, and improving AMR by mutually complementing individual differences. To our knowledge, no studies have yet reported an association between changing endoscopist and ADR. As CRC screening has become more common, opportunities to undergo colonoscopy are increasing. The detection of precancerous lesions missed in the previous colonoscopy in the second or subsequent colonoscopies will improve prognosis. Thus, the purpose of this study was to verify whether changing the endoscopist for repeat colonoscopies would improve the detection rate of missed lesions in the second colonoscopy.

## Methods

### Study design and Population

We retrospectively reviewed the database of the Omori Red Cross Hospital and extracted the data of 8093 consecutive patients who underwent colonoscopies from January 1st 2018 to June 30th 2021. Written informed consent was obtained from all patients prior to colonoscopy. To assess lesions that were not detected in the last colonoscopy, data from repeated total colonoscopies of the same patients within a limited time frame were collected. Previous studies have reported that retrospective data of short-term repeat colonoscopies, such as between 1 and 4 months, are considered theoretically practicable and comparable to those of tandem colonoscopies [14–16]. Therefore, to minimize the possibility of newly emerging lesions, only the data for repeat colonoscopies performed twice within a span of 3 months were analyzed. At our hospital, when colorectal lesions are detected, we strictly evaluate whether cold snare polypectomy (CSP) or endoscopic mucosal resection (EMR) should be performed. Although small adenomatous lesions considered to be an indication for CSP are currently resected, if outpatients are found to have large lesion considered an indication for EMR, they are usually admitted and treated on another day. At our institution, a second endoscopy for EMR on the indicated lesion was performed within approximately three months, at which time the colon was screened again, including other sites. This regime allowed for the design of this study.

### Colonoscopy Procedure

As bowel preparation, the patients ingested 2 L of polyethylene glycol electrolyte solution prior to colonoscopy. Bowel preparation quality was assessed using the Boston bowel preparation scale (BBPS) [17]. According to previous reports on BBPS and sufficient bowel preparation, we defined inadequate bowel preparation as colons with a BBPS less than 2 for any segment and sufficient bowel preparation as a colon with a total BBPS of 6 or more [17, 18].

Colonoscopies were performed using an Olympus colonoscope (EVIS LUCERA 260 series; PCF-Q260AI, CF-Q260AI, PCF-Q260AZI, PCF-Q260L, or EVIS LUCERA ELITE 290 series; CF-HQ290ZI, PCF-H290I, PCF-H290ZI; Olympus, Tokyo. Japan) with carbon dioxide insufflation. Colorectal observations were performed using white-light images with the same structural enhancement and color settings. Narrow band imaging was used only after polyp detection for qualitative diagnosis of the polyps. A tip attachment was placed on the endoscope in all cases (MAJ-1990 and MAJ-1991; Olympus). Conscious sedation with midazolam (1–5 mg) and/or pethidine hydrochloride (17.5–35 mg) was administered at the beginning of the procedure, at the discretion of the endoscopist. Intravenous antispasmodics (glucagon or scopolamine butylbromide) were administered before or during the procedure, as required.

In our hospital, a trainee was defined as an endoscopist with experience performing less than 200 colonoscopies. Procedures performed by trainees were always followed by at least one attending endoscopist on site, irrespective of whether they were the initial or second colonoscopies.

### Data Collection and Definition

All colonoscopy records were retrospectively reviewed and compared by other endoscopists twice within 3 months. We included only total colonoscopies, which were defined as colonoscopies that achieved cecal intubation, in the analysis. Cecal intubation was verified by the identification of the appendiceal orifice and ileocecal valve. To measure the quality of the initial colonoscopy, the polyps per colonoscopy (PPC) were calculated, which correlates with ADR and is considered an alternative indicator of ADR [19]. Subsequently, polyps identified in the second colonoscopy which had not been identified in the initial endoscopy were defined as missed polyps. Among the missed polyps, those that were pathologically diagnosed as adenomas or carcinomas were defined as missed lesions. The exclusion criteria were as follows: inadequate bowel preparation (BBPS < 2 for any segment), active inflammatory bowel disease, cases in which the entire colon could not be observed, such as collecting lesions, and inability to evaluate all polyps from the record (the cases in which sufficient information on polyp location or form was available for all polyps from the record).

Endoscopists were instructed to measure polyps using the size of the snare catheter or snare diameter. Lesions were classified into three groups based on size; diminutive (1–5 mm), medium (5–9 mm), and large (10 mm or larger). The index lesion was defined as an adenomatous lesion 10 mm or larger in size, or high-grade dysplasia or invasive carcinoma.

The 290 series scopes, with a 170-degree viewing angle, were defined as high-resolution scopes, and the 260 series scopes, with a 140-degree viewing angle, were defined as standard-resolution scopes. In this study, to assess the exact observation time of the colon, we considered the withdrawal time separately from the time of examination and treatment of lesions. Therefore, the withdrawal time was defined as the time from the initiation of cecal inspection to the time when the colonoscope was withdrawn from the anus, excluding the time for observing, staining, and removing the lesion; this was measured using the timer on the endoscopic images. As a previous report indicated that a withdrawal time of 6 min or more was related to increased ADR, a sufficient withdrawal time was defined as that of 6 min or more [20].

### Outcome measures

The patients and procedures were divided into two groups based on whether the two examinations were performed by the same or different endoscopists. The decision of the endoscopist was left to an outpatient gastroenterologist. The primary outcome was the missed lesion detection rate (MLDR), which was calculated as the number of patients with at least one missed lesion, divided by the total number of patients included in the analysis. The secondary outcome was the missed lesions per colonoscopy (MLPC), calculated as the number of missed lesions divided by the total number of patients in both groups. The following factors that were possibly associated with MLDR were also compared between the two groups: age, sex, body mass index, sedation, antispastic, and scope usage, withdrawal time, and bowel preparation. In the present study, all total colonoscopies were included, regardless of the history of previous colonoscopy or treatment for colorectal lesions.

### Statistical analysis

Continuous variables were summarized as the mean ± standard deviation (SD), or median and range. Categorical variables were summarized as frequencies (%). The significance of the differences in variables between groups was determined using the chi-square test for categorical variables and Student’s t-test and Mann-Whitney U test for continuous variables, as appropriate. Multiple logistic regression analysis was performed for the variables considered to be significantly correlated with MLDR using a univariate model to confirm the factors independently associated with the detection of missed lesions. All data were analyzed using JMP pro 15.0 (SAS Institute Inc., Cary, North Carolina, USA). Statistical significance was set at P < 0.05. This study was approved by the Institutional Review Board of the Omori Red Cross Hospital (approval number:22–43), and was performed in accordance with the Declaration of Helsinki.

## Results

### Study flow

**Fig. 1:**
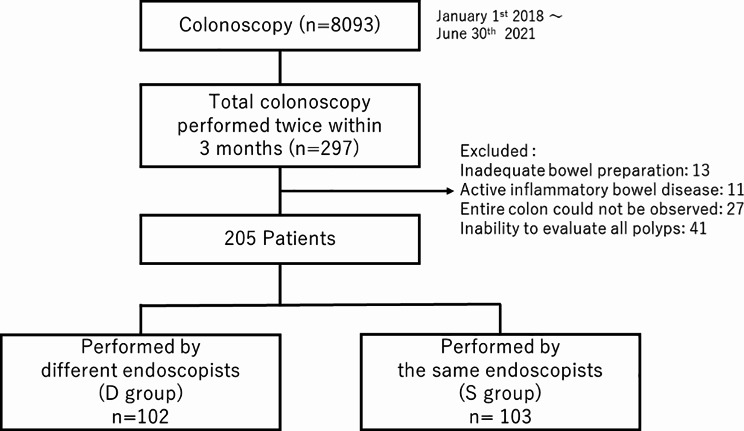
Flowchart of patient enrollment

The flowchart of the study is shown in Fig. 1. Between January 2018 and June 2021, 8093 coloscopies were performed, and 297 patients were considered eligible for the study. After excluding 92 patients, 205 eligible patients were analyzed. Of these 205 cases, 102 were performed by a different endoscopist (group D), and the other 103 cases were performed twice by the same endoscopist (group S). Of the 19 endoscopists involved in this study, 19 endoscopists including 5 trainees, belong to the D group, and 16 endoscopists, including 4 trainees, belong to the S group, with some duplication. The total quality of the initial colonoscopy was considered to be adequate with 3.90 (95% confidence interval (CI); 3.5–4.2) of PPC, which is higher than previously reported 2.2 as PPC among positive colonoscopies [21]. Polyps detected during the initial colonoscopy, but not removed, were resected during the second colonoscopy. Five cases included lesions that were detected in the initial colonoscopy which could not be detected in the second colonoscopy. Of these five patients, two were performed by the same endoscopist, and three by different endoscopists. However, since this study targeted lesions detected for the first time in the second colonoscopy, these cases were also included in the analysis.

### Patient characteristics

The patient characteristics in each group are shown in Table 1. No statistically significant differences were observed between the two groups in terms of age, sex, body mass index. In comparison to the initial and second colonoscopies, the proportion of trainee inspection (17.6% vs. 15.6%, P = 0.60), sufficient bowel preparation (5.4% vs. 4.4%, P = 0.64), and sufficient withdrawal time (6.8% vs. 11.7%, P = 0.09) was similar.

**Table 1:** Clinical characteristics of the study participants

Characteristics of the patients	D group	S group	*p* value
Number of patients (Total: 205)	102	103	
Age, (mean ± SD)	69.0 (11.0)	66.2 (11.4)	0.08
Sex, (M:F)	72:30	77:26	0.53
Body mass index, (kg/m^2^; mean ± SD)	23.2 (3.98)	23.7 (3.88)	0.38

D group, different endoscopist groups; S group, same endoscopist group; SD, standard deviation; 95% CI, confidence interval

### Outcomes of the second colonoscopies in both groups

As a prerequisite for comparing outcomes of second colonoscopies, no significant differences were found in the initial colonoscopy conditions in terms of the proportions of high-resolution scope usage, trainee inspections, sufficient bowel preparation, sufficient withdrawal time, midazolam usage, pethidine hydrochloride usage, and antispasmodic usage between groups D and S [Table 2]. Additionally, the PPC of the initial colonoscopy was also similar between both groups (4.15 vs. 3.65, P = 0.15). Table 3 shows a comparison between the two groups during the second colonoscopy. The primary endpoint, MLDR, was significantly higher in the D group (61.8% vs. 31.1%, P < 0.0001), as was the secondary endpoint, MLPC (1.29 vs. 0.60, P < 0.0001). Conversely, the proportion of trainee inspection was higher in the S group (9.8% vs. 20.4%, P = 0.03) and pethidine hydrochloride and the high-resolution scope was more often used in the D group (57.8% vs. 35.9%, P = 0.002). Scope change rate from standard to high-resolution scope between the index colonoscopy and the second colonoscopy was higher in the D group (34.3% vs. 14.6%, P = 0.001), although the rates from high-resolution to standard scope was similar (7.8% vs. 12.6%, P = 0.26).

**Table 2:** Comparison of the initial colonoscopy conditions between the two groups

Outcomes	D group	S group	*p* value
Number of patients (Total: 205)	102	103	
High resolution scope, n (%)	33 (32.4)	35 (34.0)	0.88
Performed by a trainee, n (%)	15 (14.7)	21 (20.4)	0.36
BBPS ≥ 6, n (%)	99 (97.1)	95 (92.2)	0.21
Withdrawal time > 6 min, n (%)	96 (94.1)	95 (92.2)	0.78
Total withdrawal time, median (IQR)	13.5 (10.4–17.4)	13.6 (9.3–17.4)	0.72
Midazolam usage, n (%)	88 (86.2)	96 (93.2)	0.11
Pethidine hydrochloride usage, n (%)	65 (63.7)	54 (52.4)	0.12
Antispasmodic usage, n (%)			0.88
Scopolamine butylbromide	63 (61.8)	67 (65.1)	
Glucagon	33 (32.4)	30 (29.1)	
None	6 (5.9)	6 (5.8)	
Polyps per colonoscopy (95%CI)	4.15 (3.60–4.71)	3.65 (3.10–4.19)	0.15

D group, different endoscopist group; S group, same endoscopist group; BBPS, Boston Bowel Preparation Scale; IQR, interquartile range; 95% CI, confidence interval

**Table 3:** Comparison of the results of second colonoscopy between the two groups

Outcomes	D group	S group	*p* value
Number of patients (Total: 205)	102	103	
High resolution scope, n (%)	59 (57.8)	37 (35.9)	0.002
Scope change (the initial CS → the second CS)			
High-resolution → Standard	8 (7.8)	13 (12.6)	0.26
Standard → High-resolution	35 (34.3)	15 (14.6)	0.001
Performed by a trainee, n (%)	10 (9.8)	21 (20.4)	0.03
BBPS ≥ 6, n (%)	95 (93.1)	101 (98.1)	0.09
Withdrawal time > 6 min, n (%)	91 (89.2)	90 (87.4)	0.68
Total withdrawal time, median (IQR)	11.8 (8.0-15.5)	11.8 (8.3–16.7)	0.94
Midazolam usage, n (%)	92 (90.2)	97 (94.2)	0.29
Pethidine hydrochloride usage, n (%)	30 (29.4)	52 (50.5)	0.002
Antispasmodic usage, n (%)			0.13
Scopolamine butylbromide	70 (68.6)	68 (66.0)	
Glucagon	32 (31.4)	31 (30.1)	
None	0 (0)	4 (3.8)	
Missed lesion detection rate (%)	61.8	31.1	< 0.0001
Missed lesions per colonoscopy (95%CI)	1.29 (1.00-1.58)	0.60 (0.39–0.81)	< 0.0001

D group, different endoscopist group; S group, same endoscopist group; CS, colonoscopy; BBPS, Boston Bowel Preparation Scale; IQR, interquartile range; 95% CI, confidence interval

The characteristics of the missed lesions are shown in Table 4. No significant differences were observed in terms of tumor location, size, or morphology. Although no cases of missed high-grade adenoma or adenocarcinoma were found in the same endoscopist group, there were 6 cases (4.6%) in the D group. Focused on the missed index lesions, those proportion was significantly higher in the D group (22.1% vs. 7.9%; P = 0.01).

**Table 4:** Clinicopathologic characteristics of the missed lesions in both groups

Characteristics of the missed lesions	D group	S group	*p* value
Number of lesions (Total: 194)	131	63	
Tumor location, n (%)			0.57
Right	40 (30.5%)	24 (38.1%)	
Transverse	60 (45.8%)	25 (39.7%)	
Left	31 (23.7%)	14 (22.2%)	
Tumor size			0.09
Diminutive (≤ 5 mm)	88 (67.1%)	47 (74.6)	
Medium (6–9 mm)	17 (12.3%)	11 (17.5)	
Large (≥ 10 mm)	26 (19.9%)	5 (7.9)	
Morphology			0.97
Flat	85 (64.9%)	42 (66.7%)	
Sessile	44 (33.6%)	20 (31.8%)	
Pedunculated	2 (1.5%)	1 (1.6%)	
Pathology			0.08
High grade adenoma, Tis (M)	6 (4.6%)	0 (0)	
Low grade adenoma	114 (87.0%)	61 (96.8%)	
SSL	11 (8.4%)	2 (8.4%)	
Index Lesion	29 (22.1%)	5 (7.9%)	0.01

Right, Cecum and Ascending colon; Left, Descending and Sigmoid colon and rectum; S group, same endoscopist group; D group, different endoscopist group; SSL, sessile serrated lesion; Index Lesion, adenomatous lesion ≥ 10 mm in size or high-grade dysplasia or invasive carcinoma

### Associated factors for MLDR

In multivariate analysis, the superiority of the different endoscopist groups over the same endoscopist group regarding MLDR was maintained after adjusting for high-resolution scope usage, scope change (from standard to high-resolution scope), trainee inspection, pethidine hydrochloride usage, sufficient bowel preparation (BBPS ≥ 6), and sufficient withdrawal time (withdrawal time > 6 min) (odds ratio, 3.38; 95%CI, 1.81–6.30). MLDR was also significantly associated with sufficient withdrawal time (odds ratio, 3.10; 95%CI, 1.12–8.61) [Table 5].

**Table 5:** Factors associated with missed lesion detection rate identified on multivariable analysis

Factors	OR		95% CI	*p* value
High resolution scope	1.47		0.68–3.19	0.32
Scope change (Standard → High-resolution)	0.70		0.29–1.67	0.42
Not performed by trainee	2.00		0.81-5.00	0.13
Pethidine hydrochloride use	0.85		0.45–1.60	0.61
BBPS ≥ 6	1.63		0.38–7.07	0.51
Withdrawal time > 6 min	3.10		1.12–8.61	0.03
Performed by different endoscopists	3.38		1.81–6.30	0.0001

BBPS, Boston Bowel Preparation Scale; OR odds ratio; 95% CI, confidence interval

## Discussion

In the present study, in patients undergoing repeat colonoscopy, both the detection rate and number of missed lesions were significantly higher when performed by different endoscopists than by the same endoscopist. Furthermore, multivariate analysis showed that the use of different endoscopists was independently associated with improved missed lesion detection rate.

Although ADR is now recognized as a representative quality indicator for colonoscopy, some limitations have been pointed out, such as the fact that missed lesions are not evaluated. As an indicator specific to missing, the AMR has been noted and estimated to be approximately 20% [6, 14], indicating that approximately 20% of precancerous lesions can be missed by conventional screening colonoscopy. The importance of colonoscopy in reducing the risk of CRCs is widely recognized, and it is becoming less common to perform colonoscopy only once in a lifetime [22]. If a patient undergoes a repeat colonoscopy, it is desirable to recover the missed lesions during the second and subsequent examinations.

Previous reports have described modifying instrument and endoscopist factors as ways to reduce the number of missed lesions. The superiority of endoscopes with auxiliary techniques, linked color imaging, and blue laser imaging over conventional colonoscopy for polyp detection and less missing is considered an instrumental factor [9, 23, 24]. Colorectal observations were performed in both groups using the same settings. Therefore, these factors appear to have little influence on the results. Regarding endoscopist factors, Kumar et al. indicated that shorter withdrawal time is associated with more missed polyps [25]. Focusing on an endoscopist’s fatigue, some reports have investigated whether there are differences in lesion detection between morning and afternoon colonoscopies; however, the answer remains controversial [26–29]. However, few reports have focused on endoscopist habits, such as manipulating the scope and visual gaze patterns. Although artificial intelligence could be an option to reduce the bias caused by these habits, it is limited in that it cannot recognize information which not on the screen. Therefore, we focused on the effects of changing the endoscopist to compensate for individual differences in scope manipulation and gaze patterns. Indeed, a study using eye-tracking technology showed that the visual gaze pattern differs among endoscopists even in the same recorded colonoscopy video [27]. In addition, several patterns were confirmed in scope manipulation according to the screen-based analysis. These studies indicated the existence of individual differences [28]. The correlation between polyp detection rate, visual gaze patterns, and scope manipulating patterns has also been indicated [29], suggesting that these individual differences may play a role in the polyp detection rate. In this study, different endoscopists performing repeated colonoscopies were identified as the independent factor improving MLDR. Compensating for these individual differences by switching endoscopists may be one of the factors that contributed to the results of this study. To the best of our knowledge, this is the first report to confirm the superiority of repeat colonoscopies by different endoscopists in recovering missed lesions.

In the present study, colonoscopy experience was not significantly associated with missed lesions. Previous studies have indicated that trainee participation in screening colonoscopy does not affect the ADR [30, 31]; therefore, the same could be applied to missed lesion detection rates. Furthermore, an attending endoscopist should always perform follow up when trainees perform colonoscopy at our institution. The presence of a second observer may also be a factor contributing to the lack of a significant association between trainee participation and the missed lesion detection rate. Observation by a trainee alone, without a supervisor, is preferable to accurately examine individual differences in endoscopic quality. In clinical settings, most institutions rule that trainee observations should be performed by attending endoscopists to reduce the number of missing lesions and the risk of complications. Therefore, the concept of this study is acceptable.

Sufficient withdrawal time was also an independent factor associated with missed lesion detection rates in this study. The association between withdrawal time and ADR has already been reported [17], and has been shown to be higher when the withdrawal time is 6 min or longer. Another study reported a lower AMR with a withdrawal time of 6 min compared with 3 min, suggesting that a short withdrawal time may also be related to missing lesions [25]. Based on these reports, it is considered acceptable that sufficient withdrawal time in the second colonoscopy was associated with a higher missed lesion detection rate in the present study.

Our results were strengthened by the per-polyp analysis, which showed a higher proportion of index lesions among the overall missed lesions in the D group. Furthermore, six cases of high-grade dysplasia and cancer were seen in the D group, in contrast to zero cases in the same endoscopist group. Among the missed lesions, clinically significant lesions that could lead to PCCRC were detected more often by different endoscopists, demonstrating the clinical relevance of our study.

Despite its strengths, our study has several limitations. First, this study was limited by the single-center retrospective design, which may have introduced selection bias, and the small sample size. In addition, there were some differences in background between the two groups. To minimize the impact of these differences, we performed a multivariate analysis incorporating these factors, and different endoscopists were independently associated with improving MLDR. Therefore, we believe that this research is valuable for discussing missing lesions. Second, missed lesions may have persisted even during the second colonoscopy procedure. Third, many patients in our study had at least one adenoma indicated for EMR; among these patients, the chances of missing lesions may be higher than those in patients with no lesions. MLDR in our study was much higher than AMR previously reported [6, 7, 11]. Previous study reported that patients with large lesions are at high risk of metachronous adenoma [32], therefore the patients involved in this study might be at high risk of metachronous adenoma. In addition, polyp number in previous colonoscopies was also considered to be associated with metachronous colorectal neoplasia [32]. The PPC of our study in the initial colonoscopy was 3.90, which means the patients in our study had more polyps than the natural population. This also supports the possibility that the patient in our study might be at high risk of metachronous adenomas. For these reasons, MLDR may be estimated to be higher than that in the normal population. Considering these limitations, we plan to conduct a future multicenter prospective study to confirm our results.

## Conclusions

In conclusion, our study showed a significant improvement in the detection of missed lesions when performed by different endoscopists. When repeated colonoscopies are performed on the same patient, it is preferable that a different endoscopist perform the second colonoscopy, to increase the chances of identifying any missed lesions.

## Data Availability

The datasets generated and analysed during the current study are not publicly available due to individual privacy could be compromised but are available from the corresponding author on reasonable request.

## List of abbreviations

CRC: colorectal cancer
ADR: adenoma detection rate
PCCRC: post colonoscopy colorectal cancer
AMR: adenoma miss rate
BBPS: Boston Bowel Preparation Scale
PPC: polyps per colonoscopy
MLDR: missed lesion detection rate
MLPC: missed lesion per colonoscopy
CSP: Cold snare polypectomy
EMR: Endoscopic mucosal resection
95% CI: 95% confidence interval
SD: standard deviation
